# Effects of Aqueous Extracts from Wheat Bran Layers on the Functional Properties of Wheat Starch and Gluten

**DOI:** 10.3390/foods14111988

**Published:** 2025-06-04

**Authors:** Bingbing Wu, Chunlei Yu, Zhongwei Chen, Bin Xu

**Affiliations:** School of Food and Biological Engineering, Jiangsu University, Zhenjiang 212013, China; wubingbing0910@163.com (B.W.); xxgzyxzh@163.com (C.Y.); taotaoxu@126.com (B.X.)

**Keywords:** wheat bran, aleurone layer, aqueous extract, rheological properties, starch gelatinization

## Abstract

Wheat bran (WB) is rich in bioactive compounds, but its incorporation into food products often negatively affects dough properties. The soluble components in WB, including polysaccharides, minerals, and proteins, exhibit significant variations across different bran layers and may dissolve and interact with flour components during food processing, affecting dough properties. This study aims to investigate the influence of aqueous extracts from different WB layers (aleurone layer, AL; non-aleurone layer, NAL) and their components on the functional properties of wheat starch and gluten. The results indicate that the AL-rich fraction yielded a higher extract content (30.6%) compared to the NAL-rich fraction (15.1%), attributable to the higher cellular content in the AL. Both the extracts and residues from AL and NAL significantly lowered the denaturation temperature of wheat gluten. The aqueous extracts reduced the storage (G′) and loss (G″) moduli of wheat gluten, primarily attributed to the effect of polysaccharide components, whereas the protein and ash fractions elevated the G′ and G″ at suitable dosages. The extracts elevated the gelatinization temperature of starch, but reduced enthalpy (ΔH). Moreover, the pasting viscosity of starch with WB extract decreased due to the combined effects of protein and ash fractions. These findings provide insights into the roles of water extracts from different WB layers and their components in modulating wheat-based product quality. This study also offers a theoretical basis for optimizing WB utilization in foods, thus providing a theoretical foundation for promoting whole-wheat foods or foods containing WB.

## 1. Introduction

Wheat bran (WB) is the main by-product of wheat flour milling, with a global annual production exceeding 150 million tons [1]. WB is well-recognized for its potential health benefits in preventing chronic diseases, such as obesity, type 2 diabetes, cardiovascular diseases, and some cancers, due to its higher content of dietary fiber and bioactive components [2,3]. Despite its potential as a potential source for processing healthy foods, WB remains predominantly used in animal feed due to two major limitations for food applications [1]. First, as the outer layer of wheat grain, WB contains various contaminants, including microorganisms, pesticide residues (neonicotinoids), and heavy metals (Cd, Pb), which raise safety concerns [1,4,5]. Second, certain WB components negatively affect wheat flour processing properties, including dough-mixing and rheological characteristics. These effects stem from interactions between WB components (particularly cell walls and soluble compounds) and wheat starch/gluten during processing [6,7].

Wheat bran consists of three distinct layers: an aleurone layer (AL), outer pericarp (OP), and an intermediate layer (IL) combining inner pericarp, testa, and hyaline layer [8]. These layers vary in structure, chemical composition, and physical properties [9]. OP and IL are primarily composed of a cell wall, while the AL contains ~40% cell wall and ~60% cell content [1]. The cell wall composition differs substantially between layers. The cell walls of OP and IL are composed by cellulose (33%), arabinoxylans (AX, ~60%), and lignin (5%), while AL cell walls are mainly composed of AX (~65%) and β-glucan (~29%), with small amounts of cellulose (~2%) [10,11]. As the only layer with cell content, the AL is rich in proteins, minerals, B vitamins, and lipids [8,12], which are concentrated in its cell content. Components derived from WB cell walls or cellular contents will interact with starch and gluten, thereby exerting differential effects on dough properties and food quality attributes [1,6,7]. Therefore, these differences in structure and chemical composition give the WB layers different properties for processing foods. For instance, the insoluble dietary fiber (mainly cell wall) in OP and IL negatively impacts wheat products by disrupting gluten network formation and reducing product volume [13]. In comparison, soluble components, such as water extractable AX (WEAX) and proteins can improve dough properties by increasing water absorption and viscosity [14]. It has been shown that WEAX could promote gluten aggregation, enhance network stability, and facilitate uniform gluten matrix formation [14,15,16]. The addition of WB proteins, which are mainly from AL, especially the AL cell content [17], does not significantly affect the baking characteristics of bread and noodles, imparting a more attractive color and increasing the total content of essential amino acids in the food [18,19]. Other water-soluble components in WB, such as monosaccharides, minerals, phytic acid, and B vitamins, may also influence the processing the wheat flour. Consequently, understanding the effects of the soluble components from WB is crucial for improving the quality of whole-wheat food.

The influence of soluble fraction from WB has been studied in a previous study [20], in which the soluble components were extracted using different methods, such as water, and subcritical water with or without enzymatic treatment [20]. The water-extractable components include WEAX, β-glucan, minerals, proteins, phenolic acids, etc. [21,22,23]. The extracts yield varied from 15% to 58% due to the differences in particle sizes, milling processes, and extraction processing of WB [20,23,24,25]. It has been found that WB extract (WB-E) could not only improve the processing characteristics of the products, but also endow their nutritional value. The addition of extracts from WB improves the viscoelasticity of the dough, particularly its extensibility; however, WB-E does not significantly affect the secondary structure of gluten [24]. Bread containing WB-E showed a significant increase in volume and specific volume, resulting in a small and uniform crumb structure [24]. Currently, some studies have investigated the impact of WB on starch in wheat flour [20,26,27,28]; however, existing studies on aqueous extracts from WB predominantly focus on bulk extracts from whole WB or single compound, with limited attention to layer-specific aqueous extracts and their synergistic interactions with wheat flour components. Additionally, WB consists of the layers with varying chemical compositions and physical structures, leading to differences in extraction components, yields, and effects on starch and gluten properties. The compositional disparities in the aqueous extracts from AL and NAL may lead to divergent impacts on starch gelatinization, gluten network formation, and food qualities.

In this study, the effects of water extracts from different WB layers, i.e., AL and NAL, on wheat starch and gluten were investigated. First, high-purity AL and NAL were obtained using electrostatic separation. The extracts from the WB layers were obtained via aqueous extraction and purified into protein-rich, polysaccharides-rich, mineral-rich fractions. Finally, the impact of these fractions on the thermal and rheological properties, as well as the pasting characteristics, of starch and gluten were investigated. The results of this work will provide a new understanding the effects of adding WB on dough and facilitate the production of high-quality whole-wheat flour foods.

## 2. Materials and Methods

### 2.1. Materials

The WB and the wheat flour was purchased from Wudeli Flour Group Co., (Handan, China). The Total Starch Assay Kit (K-TSTA) was purchased from Megazyme (Bray, Ireland). The chemicals sodium chloride, petroleum ether, anhydrous ethanol, and trifluoroacetic acid were purchased from the Sinopharm Group (Beijing, China).

### 2.2. Sample Preparation

The preparation of the samples for this study included three main steps. First, the aleurone (AL)-rich fraction and non-aleurone-rich fractions were obtained using centrifugal impact milling and triboelectric separation; then, aqueous extracts of the AL and NAL were prepared using water extraction; lastly, the different components, including crude proteins, polysaccharides, and ash, were isolated using dialysis and concentration. The detailed procedures are shown as follows.

#### 2.2.1. Separation of AL and Non-AL Fractions

**Figure 1:**
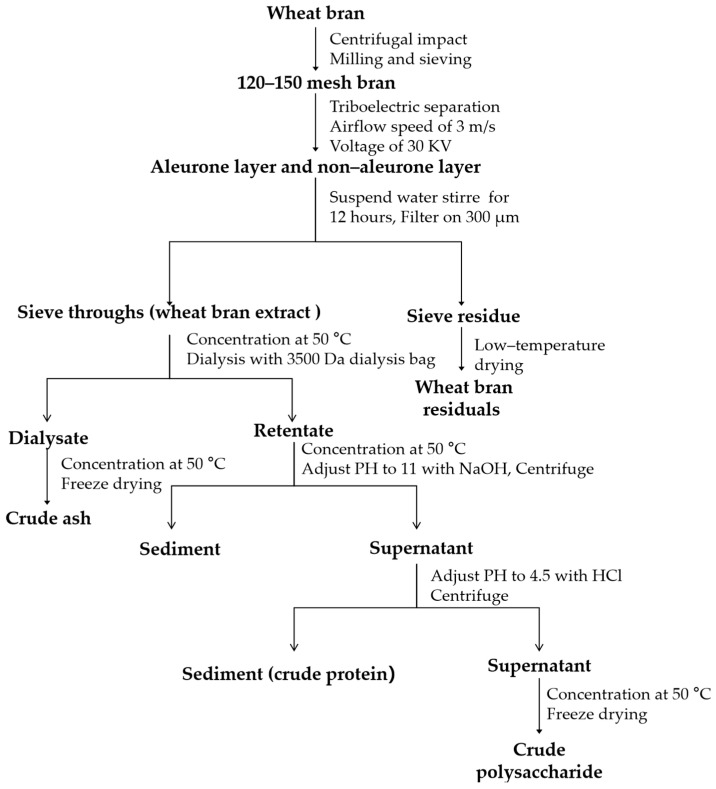
Procedure for the extraction and separation of water extracts from different WB layers.

As shown in Figure 1, WB was ground using centrifugal impact milling (CIM) and sieving to obtain the fraction with dissociated WB layers according to the method of Chen et al. [29]. During the CIM, the temperature of the crushing chamber was maintained below 15 °C using cold airflow, and the bran particles were transported out by airflow. The bran fraction with a particle size of 120–150 mesh (100–150 μm) was used as the starting material for electrostatic separation. A lab-scale triboelectric separation (TES) system was employed to obtain AL-rich and non-AL-rich fractions based on the method of Chen et al. [29,30]. During TES, the airflow speed was set to 3.0 m/s, and the voltage was 30 kV. The AL and non-AL particles were charged by the particle-wall and particle-particle collisions and then were enriched by the electrostatic force in the electric field. Three vessels were used at the bottom of separation chamber to recover the bran fractions during TES. The bran fragments between the electrodes were collected in the middle vessel, and the bran fragments on the positive and negative electrodes were collected in the other two vessels. After the first step of TES, the bran fraction in the middle vessel and the vessel near the negative electrode were used as the starting material for the subsequent enrichment of non-AL (OP and IL) and AL, respectively, in the second step of the TES. The AL-rich fraction (7.9% yield) and non-AL-rich fraction (17.2% yield) were obtained after three steps of TES and sealed in a desiccator before further analysis.

#### 2.2.2. Preparation of Extracts from WB Layers

The AL and non-AL fractions were mixed with distilled water at a ratio of 1:10 (*w*/*v*). The mixture was stirred at 25 °C for 12 h and then filtered through a 300 μm mesh. The residue was centrifuged (4000 rpm for 10 min), and the supernatant was concentrated using rotary evaporation (at 50 °C) and then freeze-dried to obtain the WB layer extracts. The residue from the filtration and the precipitate from the centrifugation were combined and dried to obtain the WB-E.

#### 2.2.3. Separation of Major Components in WB-E

The aqueous extract of WB layers was concentrated and transferred into a dialysis bag with a molecular weight cutoff of 3500 Da. Dialysis was conducted for 24 h in deionized water at 25 °C, with the water changed once after 3 h. The solution outside the dialysis bag was concentrated using rotary evaporation at 50 °C and then freeze-dried to obtain crude ash and sugar. The retentate in the dialysis bag was first adjusted to pH 11 by adding 1.0 M sodium hydroxide solution maintained at 50 °C and stirred for 1.5 h, before centrifugation at 5000× *g* for 30 min to collect the supernatant. Then, the supernatant was acidified with 1 M hydrochloric acid until the pH reached around 4.5 to precipitate proteins [31]. It was appropriately concentrated and then freeze-dried to obtain crude polysaccharides [31]. The extraction and separation of solubilized components from WB layers are illustrated in Figure 1.

#### 2.2.4. Extraction of Starch and Gluten Proteins

Wheat flour, sodium chloride, and distilled water were mixed at a ratio of 50:1:25 (*w*/*w*/*v*) to form a dough, which was then placed in a chamber (30 °C, 85%) for 30 min. The dough was then washed until the wash water was clear, and the resulting gluten balls were freeze-dried. The wash water containing starch was passed through a 150-mesh screen and allowed to stand overnight. The upper layer of water was discarded, and the precipitated slurry was centrifuged at 4000 rpm for 10 min. The precipitate was washed twice with distilled water and dehydrated with anhydrous ethanol, and then air-dried to obtain starch. The dried wheat starch and freeze-dried gluten protein were ground through an 80-mesh screen and set aside for later use.

#### 2.2.5. Preparation of Blended Flour

Blended flour with wheat bran extract (WB-E) (including gluten proteins and starch) was prepared by adding extracts at concentrations of 3%, 6%, and 9% back to gluten proteins/starch. WB layers and their residuals (WB-R) were added back to wheat gluten proteins/starch at concentrations of 10%, 15%, and 20% and were then mixed uniformly using a three-dimensional mixer (JHX4-DM, Zhejiang New Century Crushing Co., Ltd., Ruian, China).

Since approximately 20% of reducing sugars were still present in the crude ash, and the majority of reducing sugars in bran are glucose, experiments were designed to investigate the effects of glucose on the properties of gluten and starch. This aimed to clarify the influence of ash on the characteristics of gluten and starch. In preliminary experiments, the addition of bran extracts to starch and gluten did not exceed 9%, and the proportion of each major component in the extract was less than 30%. Therefore, the addition levels of each component in the bran extract were designed to 1%, 2%, and 3%.

### 2.3. Chemical Composition Analysis

The moisture, lipid, protein, and ash content of the WB layers and the extracts were determined according to AACC methods 44-15, 30-25, 46-10, and 08-01, respectively [32]. The total starch content of the bran fractions was measured using the Megazyme total starch assay kit (K-TSTA) based on the AOAC method 996.11 [33]. The total phosphorus content of the bran fractions was determined according to the AOAC official method 965.17 [33]. The total carbohydrate content was determined by the phenol–sulfuric acid method [34], and the reducing sugar content was determined by 3, 5-dinitrosalicylic acid (DNSA) colorimetric method [35]. The content of non-starch polysaccharides was obtained by subtracting the content of reducing sugar and starch from the total carbohydrate content.

Phenolic acids in the sample were released according to the method of Antoine, Lullien-Pellerin, Abecassis, and Rouau [36]. About 20 mg sample was treated with 2 M NaOH (10 mL) at 35 °C for 2 h in the dark and under nitrogen. Internal standard (3,4,5-tri-methoxycinnamic acid, 1 mg/mL, 50 μL) was added to each sample before the solution was adjusted to pH = 2.0 using 6 M HCl. The phenolic acids were extracted twice using ethyl acetate (three volumes), then the ethyl acetate phases were combined, concentrated, and dried in the presence of nitrogen. Lastly, the dry extraction was dissolved in 0.8 mL solution of water/methanol (50/50, *v*/*v*), filtered (0.45 μm), and injected (10 μL) onto an Eclipse Plus Phenyl-Hexyl column (4.6 × 250 mm, 5 μm). The linear gradient elution condition of HPLC was the same as that described by Dobberstein and Bunzel [37]. Identifications and qualification of ferulic acid and *para*-coumaric acid were accomplished by comparing the retention time of standard compounds. Ferulic acid dehydrodimers (DHD) including 8-5’-DFA, 8-O-4’-DFA, 8-5’(benzofuran)-DFA and 5-5’-DFA, and dehydrotrimer (FAt, 5-5, 8-O-4-TriFA) were identified and calculated by comparing the retention time and response factors determined.

### 2.4. Microscopic Observation

Optical and polarized light microscopy observation: WB layers were placed on a slide, covered with a cover slip with a few drops of deionized water, and observed under a bright-field microscope (BX-53, Olympus, Tokyo, Japan) equipped with a DP digital camera system. For the observation of the polarizing characteristics of cell walls in WB layer, a first-order polarizing filter (530 nm) was added, and the observation was made under a polarizing microscope.

Scanning Electron Microscopy (SEM) observation: Before observation, all bran samples were dried in an oven at 50 °C. The samples were then attached to double-sided copper-conductive adhesive and sputter-coated with gold. The sputter-coated samples were observed under a SEM (JSM-7800F, Jeol Ltd., Tokyo, Japan) at an acceleration voltage of 5.0 kV and appropriate magnification. Representative images were selected for all observations.

### 2.5. Thermal Properties

The effects of WB-E on the thermal properties of starch and gluten were determined using a differential scanning calorimeter (DSC 3500, NETZSCH Instruments, Selb, Germany) and modified according to the two methods [20,31]. Five milligrams samples containing different concentrations of WB-E (0%, 3%, 6%, 9%), WBR (10%, 15%, 20%), and major components in WB-E (1%, 2%, 3%) were weighed and added into aluminum pans with 15 μL water, sealed, and equilibrated at room temperature for at least 12 h. The equilibrated samples were heated from 20 °C to 100 °C at a rate of 10 °C/min, and the relevant temperatures were determined using TA Universal Analysis software, with data including the onset temperature (T_o_), peak temperature (T_p_), conclusion temperature (T_c_), and gelatinization enthalpy (∆H).

The impact of extracts on the thermal properties of gluten was investigated using the same method as above. The gluten samples were washed from the dough containing different concentrations of extracts and then freeze-dried and ground. Five milligrams of the sample and 15 μL water was weighed into an aluminum pan, sealed, and equilibrated at room temperature for at least 12 h. The equilibrated samples were then heated from 20 °C to 100 °C at a rate of 10 °C/min, and the denaturation temperature (T_d_) of gluten were determined using TA Universal Analysis software.

### 2.6. Dynamic Rheological Properties

The effects of extracts on the dynamic rheological properties of gluten were measured using a rotational rheometer (DHR-1, Waters China Co., Ltd., Milford, MA, USA). The dough was prepared by mixing samples with gluten and deionized water in a mass ratio of 1:1.6 and stirring for 3 min. The dough was then sealed with plastic wrap and allowed to rest at room temperature for 15 min before trimming to reduce the effect of residuals stress relaxation on the experiment. The plate was then lowered to a thickness of 1 mm and allowed to rest for 5 min before testing. First, a strain sweep test was performed in oscillatory mode to determine the linear viscoelastic region of the dough. The test parameters were set as follows: temperature 25 °C, a 40 mm diameter plate, 1 mm plate gap, scanning frequency of 1 Hz, and strain range of 0.01~1%. Subsequently, a frequency sweep test was performed on the dough at a suitable strain value within the linear viscoelastic region. The test parameters were set to a temperature of 25 °C, a 40 mm diameter plate, a scanning frequency of 0.1–10 Hz, and a strain of 0.1%. The main test results included the storage modulus (G′), loss modulus (G″), and loss tangent value (tan δ = G″/G′).

### 2.7. Pasting Characteristics

The pasting characteristics were evaluated with a Rapid Visco Analyzer (RVA) (RVA-4, Perten Instruments, Beijing, China) according to AACC method 76-21 [32]. In this analysis, wheat flour (3 g, 14% mb) was added to distilled water (25 mL). The test program was as follows: the mixture was held at 50 °C for 1 min, heated to 95 °C in 4 min and 42 s, held for 7 min and 12 s, and then cooled to 50 °C in 11 min, with a rotation speed set at 160 r/min.

### 2.8. Statistical Analysis

Each sample was measured in triplicate (*n* = 3). Data were analyzed for mean, standard deviation, and significant differences using one-way ANOVA with SPSS 27.0 (IBM Corporation, New York, NY, USA). Post hoc multiple comparison tests were performed using Duncan’s test to determine which groups differed significantly. A *p*-value of *p <* 0.05 indicated a significant difference in the data.

## 3. Results and Discussion

### 3.1. Microstructure and Chemical Composition of WB Fractions Before Water Extraction

**Figure 2:**
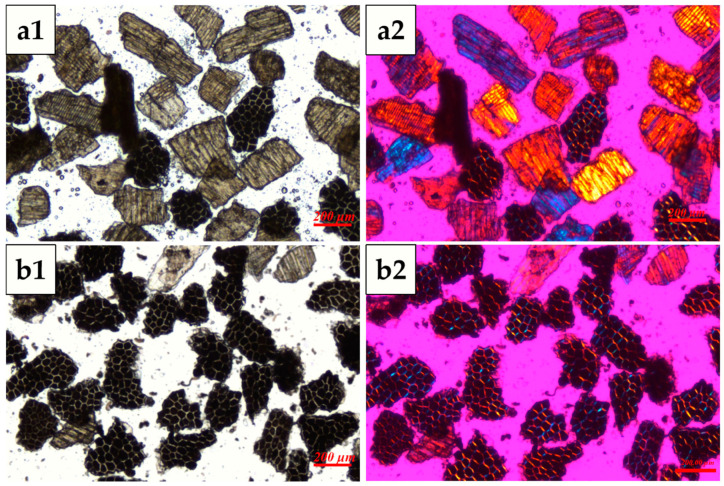
Microstructures of AL-rich and NAL-rich fractions obtained using tribo-electrostatic separation. (**a**) NAL-rich fraction, (**b**) AL-rich fraction. (**a1**,**b1**) were observed under an optical microscope; (**a2**,**b2**) were observed under a polarizing light of 530 nm.

The microstructures of AL-rich and NAL-rich fractions was shown in Figure 2. It can be found that the NAL-rich fraction was mainly composed of IN and OP, exhibiting a brown color and a certain degree of transparency under the optical microscope (Figure 2(a1)), while the AL-rich fraction mostly contained the fragments of higher opaqueness due to its high thickness and the presence of cell contents (Figure 2(b1)). This result agrees with the previous study [38]. Under the polarizing light of 530 nm, the cell walls of the OP, IN, and AL exhibited birefringence in blue and orange colors, indicating a high degree order within their cell walls. The cell walls of the NAL exhibited negative birefringence under polarized light microscopy, displaying distinct interference colors dependent on their orientation. When aligned along the northeast–southwest axis, the walls manifested orange interference tones, whereas reorientation to the southeast–northwest axis produced blue interference colors. This directional chromatic shift arises from anisotropic refractive index variations in the wall’s ultrastructure, consistent with ordered molecular arrangements (e.g., cellulose microfibrils) that generate the differential retardation of polarized light wavelengths (Figure 2(a2)). In the AL cell cluster, the cell walls exhibited positive birefringence, extending in two different directions to form a continuous network (Figure 2(b2)). The presence of birefringence in the fragments of WB layers allows for a more distinct differentiation between the AL and non-AL fragments.

As shown in Table 1, the chemical composition of WB, AL-rich, and NAL-rich fractions diverged significantly (*p* < 0.05). The AL fraction exhibited markedly higher levels of lipids, ash, phosphorus, and protein compared to both NAL and WB, reinforcing its role as a nutrient-dense compartment within the bran structure. The AL-rich fraction contained 1.5–2 times higher levels of starch, protein, ash, and lipids than the WB and NAL-rich fractions. Additionally, the phosphorus in the AL-rich fraction was 2.02 times more than that in the NAL fractions (2.18% vs. 0.93%, *p* < 0.05). Previous studies have reported that approximately 80% of the phosphorus in WB exists in the form of phytic acid and is concentrated in AL cell content [30]. Thus, the higher phosphorus content in the AL-rich fraction indicated its enrichment in AL components. Based on the biomarker method [36], the proportion of AL in the AL-rich fraction was 75.9%, whereas that in the NAL-rich fraction was about 32.4%. Furthermore, the content of ferulic acid and *p*-coumaric acid, which are predominantly localized in AL cell walls, was higher than that in the OP. In summary, the AL-rich and NAL-rich fractions exhibited distinct microstructural and compositional differences, making them suitable for subsequent water extraction and investigation.

### 3.2. Composition of Aqueous Extracts and Residuals from Different WB Layers Fractions

As shown in Table 2, the aqueous extracts and residual fractions derived from AL-rich and NAL-rich fractions exhibited significant compositional divergences (*p* < 0.05) in composition and yield. The yield of aqueous extract from the AL-rich fraction reached 30.61%, which was twice that from the NAL-rich fraction (15.08%). This disparity is attributable to the predominant origin of extracts from intracellular components, which are absent in OP and IL. Compositional divergences were observed between the extracts from AL-rich and NAL-rich fractions. Specifically, the extract from the AL fraction displayed significantly higher ash (19.51% vs. 16.09%), protein (29.41% vs. 22.61%), and lower non-starch polysaccharide content (29.89% vs. 39.04%) compared to the extract from NAL (*p* < 0.05). These variations likely arise from the differences in the structural and compositional distinctions of AL and NAL [1]. Furthermore, according to Table 1**,** the NAL-rich fraction contained about 32% AL, implying NAL extracts may retain partial components from AL. Moreover, the NAL exhibited a substantially higher proportion of cell wall (70–80%) than the AL (~30%) [1], which aligns with its elevated non-starch polysaccharides content (39.04% vs. 29.89%).

A comparative analysis of extracts and residual fractions revealed that ash content in the extracts ranged from 16.09% to 19.51%, with phosphorus levels consistently exceeding those in residual fractions. Similarly, protein content in the extracts was a higher (29.41% vs. 16.15% for the AL-rich fraction, 22.61% vs. 9.96% for the NAL-rich fraction) than the residual fractions, which agreed with a previous work [24]. Interestingly, despite the fact that lipids are predominantly localized in AL cell content [1], their concentration in aqueous extracts was markedly lower than in residual fractions [25,39], suggesting the limited extractability of lipidic components under aqueous conditions [24,25,39].

**Figure 3:**
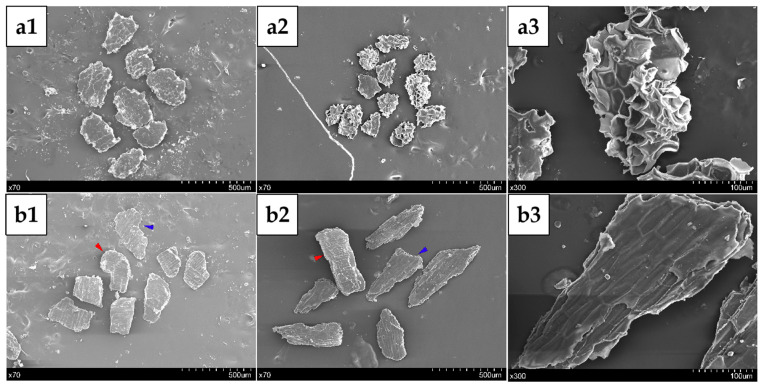
SEM micrographs of AL and NAL cell clusters before and after water extraction. (**a1**,**b1**) AL-rich fraction and NAL-rich fraction as-is; (**a2**,**a3**) residuals of AL-rich fraction after water extraction; (**b2**,**b3**) NAL-rich fraction after extraction, with magnified details. The red and blue arrows indicate the IN and OP fragments in the NAL-rich fraction, respectively.

The morphological characteristics of the residues obtained after water extraction were analyzed using scanning electron microscopy (SEM). As shown in Figure 3, the cells in AL changed from being plump and intact (Figure 3(a1)) to exhibiting structural ruptures and cavities (Figure 3(a2)). In contrast, the cells of the OP (marked by the blue arrows) and IN (marked by the red arrows) showed no significant changes (Figure 3(b1,b2)). This observation was further supported by the magnified images of the outer bran (Figure 3(b3)). Additionally, the magnified views revealed notable depressions and ruptures within AL cell clusters (Figure 3(a3)), which strongly suggests that a substantial extraction of intracellular components from the AL had occurred. This structural stability is likely attributed to their lower intracellular content and mechanically robust cell walls. The cell walls of OP and IN primarily consists of cellulose, highly cross-linked arabinoxylan (AX), and lignin [1]. Conversely, the AL contains negligible cellulose (~2%) and lignin, resulting in its weaker structural integrity and heightened susceptibility to aqueous extraction [1].

Three different fractions were isolated from WB-E. As shown in Table 3, the protein-rich fraction obtained via the isoelectric point method exhibited the highest protein purity of 78.34%. The polysaccharide-rich fraction comprised 53.44% total carbohydrates, with ash content reaching 18.06%, likely due to residual sodium ions introduced during protein precipitation. The ash-rich fraction (outer solution of the dialysis bag) contained the highest ash content (31.70%) along with 20.51% total sugar. Although the purity of these three components is not very high, their significant compositional differences could reflect the impact of polysaccharides, proteins, and ash on the properties of wheat starch and gluten.

### 3.3. Influence of WB-E on Thermal and Rheological Properties of Wheat Gluten

**Figure 4:**
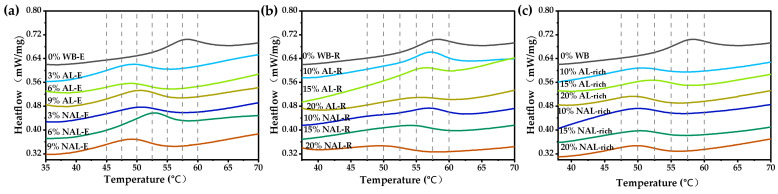
Influence of extracts and residuals from WB on the thermal properties of wheat gluten. (**a**–**c**) were thermal properties of gluten with varying contents of WB-E, WB-R, and WB layers, respectively. WB, wheat bran; AL, aleurone layer; NAL, non-aleurone layer; AL-E, extracts from AL-rich fraction; AL-R, residues of AL-rich fraction; NAL-E, extracts of NAL; NAL-R, residuals of NAL-rich fraction. 0–9% AL-E, dough made with gluten and 0–9% AL-E addition; 0–9% NAL-E, dough made with gluten and 0–9% NAL-E addition; 0–20% AL-R, dough made with gluten and 0–20% AL-R addition; 0–20% NAL-R, dough made with gluten and 0–20% NAL-R addition; 0–20% AL-rich dough made with gluten and 0–20% AL-rich fraction addition; 0–20% NAL dough made with gluten and 0–20% NAL-rich fraction addition.

To evaluate the impact of water extracts from WB and its layers on wheat gluten’s properties, WB-E was incorporated into gluten at concentrations of 3%, 6%, and 9% (*w*/*w*). As shown in Figure 4 and Table S1, both the additions of extracts (AL-E and NAL-E) and residues (AL-R and NAL-R) of WB layers significantly reduced the denaturation temperature (T_d_) of wheat gluten. Increasing the dosage of AL-E did not significantly alter the denaturation temperature of gluten, whereas the addition of NAL-E initially increased then decreased T_d_. This difference may be due to their different mechanisms for affecting gluten denaturation. The WB-E affected the denaturation of gluten protein through the interaction of minerals, AX, and phenolics with gluten, which can stabilize the gluten network, making it more resistant to heat-induced denaturation, while the residues (primarily cell wall) of WB fractions have a high water-holding capacity, which will restrict water mobility within gluten matrix and delay the denaturation. As to the effects of adding WB, WB layers (AL-rich, NAL-rich), and WB-R (AL-R and NAL-R), when the addition of AL reached 20%, the T_d_ of gluten significantly decreased, whereas 15% NAL addition elevated T_d_. The wheat gluten after adding WB-E exhibited a higher ∆H, while the samples with residues and WB showed fluctuating ∆H due to their higher amounts added.

**Figure 5:**
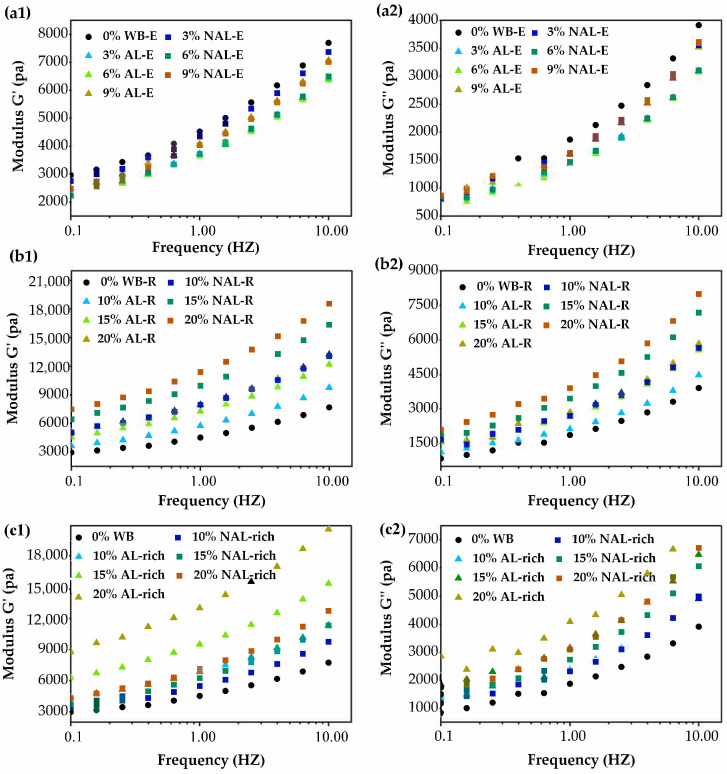
The influence of extracts and residues from WB layers on the dynamic rheological properties of wheat gluten. (**a**–**c**) were dynamic rheological profiles of gluten with varying contents of WB-E, WB-R, and WB layers, respectively. (**1**,**2**) storage modulus (G′) and loss modulus (G″) profiles, respectively. AL, aleurone layer; NAL, non-aleurone layer; AL-E, extracts from AL-rich fraction; AL-R, residues of AL-rich fraction; NAL-E, extracts of NAL; NAL-R, residuals of NAL-rich fraction.

The effects of WB-E (AL-E and NAL-E), WB-R (AL-R and NAL-R), and WB layers (AL-rich and NAL-rich) on the rheological properties of wheat gluten was illustrated in Figure 5. The gluten doughs containing WB-E exhibited lower G′ and G″ than the wheat gluten control. As the concentrations of AL-E and NAL-E increased from 3% to 9%, both the G′ and G″ decreased initially and then increased. The addition of WB and WB-R significantly enhanced the G′ and G″ relative to the control, showing dose-dependent amplification as addition levels increased. At the same addition levels, AL-R had a more significant effect than NAL-R. The enhanced viscoelasticity of gluten by WB-R may be due to the competition between gluten and cell wall for water absorption [40], and the physical hindrance to the formation of gluten network [41,42]. In brief, these results demonstrate that the WB-E (AL-E and NAL-E) and WB-R showed opposite effects on the rheological properties of wheat gluten.

**Figure 6:**
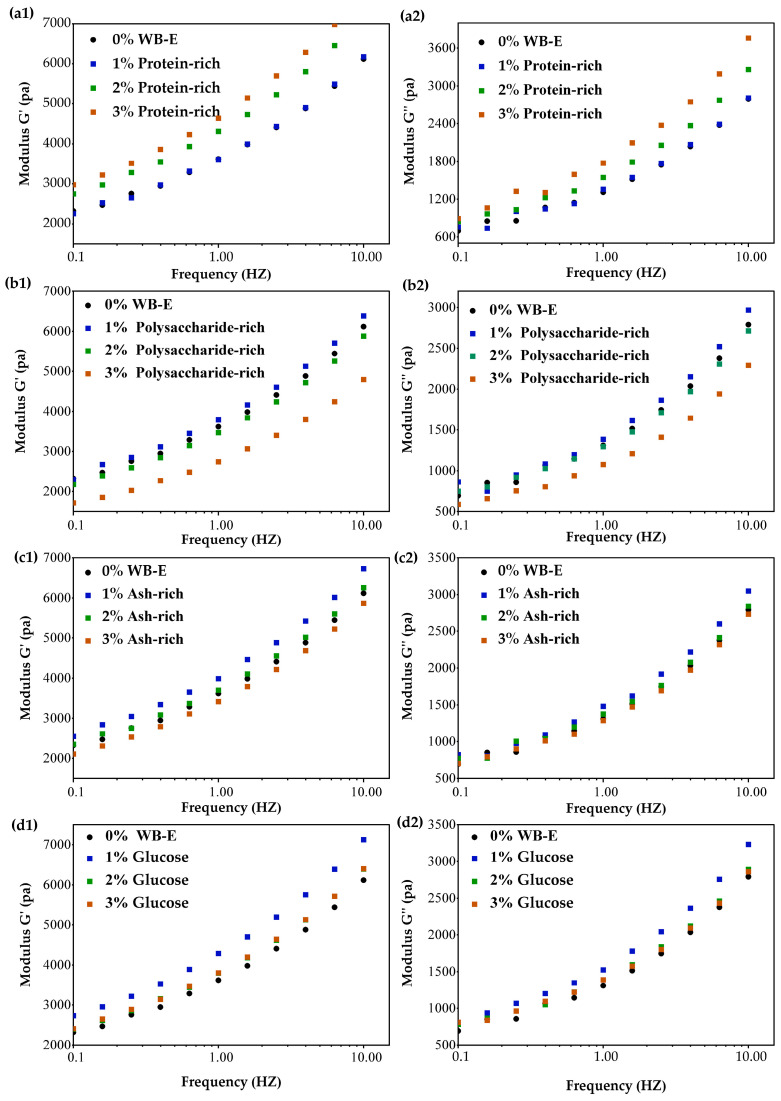
Influence of the major components in WB-E on the rheological properties of wheat gluten. (**a**–**d**) were dynamic rheological profiles of gluten with varying contents of protein-rich, polysaccharide-rich, ash-rich and glucose fractions, respectively. (**1**,**2**) storage modulus (G′) and loss modulus (G″) profiles, respectively. WB-E, wheat bran extract; 0–3% protein-rich dough made with wheat gluten and 0–3% protein-rich fraction. Similar meaning for the 0–3% polysaccharide-rich, ash-rich, and glucose fractions.

To detail the impacts of different components in extracts on rheology properties, the protein-rich, ash-rich, and polysaccharides-rich fractions were isolated and incorporated into wheat gluten. As shown in Figure 6, the addition of 1% polysaccharide-rich fraction exhibited negligible effects on gluten rheology. However, when the added amount increased to 2% and 3%, both the G′ and G″ of wheat gluten significantly decreased. This is in line with previous studies [43], indicating that WEAX weakened the dough by reducing the availability of free water for adequate gluten formation because of its hygroscopic nature. The interactions between the non-starch polysaccharides (mainly AX) and gluten occurred through non-covalent interactions [44,45]. These could disrupt the gluten network’s extensibility and aggregation kinetics, as confirmed by FTIR evidence of β-sheet reduction [16]. Concurrently, AX-induced increases in dough viscosity may impede gluten hydration, further compromising network formation. Soluble polysaccharides can interact with gluten proteins through hydrogen bonding, forming a composite network and reinforcing the gluten structure. Therefore, they can make the gluten or dough either more elastic or more extensible.

In contrast, the addition of protein-rich fraction elevated the G′ and G″ of gluten, aligning with the findings of Wang et al. [46]. In WB-E, the main proteins are albumins and globulins. While globulin-mediated viscosity enhancement (reflected in elevated G″) arises from their high-water solubility, albumin–gluten interactions via sulfhydryl-disulfide interchange reactions generally destabilize the gluten matrix. Trace bran proteins may optimize gluten hydration through improved water distribution, thereby augmenting the elastic modulus (G′).

The addition of the ash-rich fraction (1–2%) increased the G′ and G″ of gluten, likely due to mineral interactions (K, Mg, P) reducing repulsive forces between charged side chains and stabilizing the protein network [47]. Generally, in a water flour system at normal pH (6–7), the gluten proteins have net positive charges that repulse each other. A low level of salts shields the charges, allowing for the protein molecular approaching each other to from a stronger dough and higher G′ and G″ [47]. However, at 3% addition, G′ and G″ decreased, probably because excess ions over-screen charges, disrupting the delicate balance of attractive and repulsive forces within the gluten matrix [47].

The ash-rich fraction contained approximately 23% reducing sugars (mainly glucose) [24]. Glucose was added into wheat dough to verify the influence of reduce sugar on gluten rheology. It can be observed that when the amount was 1%, G′ and G″ of gluten increased, while adding amount increased to more than 2% decreased the G′ and G″. This result confirms the impact of reducing sugars in the ash fractions on the rheology of wheat gluten.

In summary, the protein and ash fractions enhanced the G′ and G″, while the polysaccharides fraction at high content reduced the rheological parameters of gluten.

### 3.4. Influence of WB-E on Thermal Properties and Pasting Characteristics of Starch

**Figure 7:**
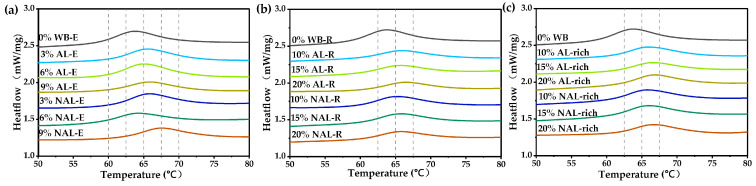
Influence of extracts and residues from WB on the thermal property of wheat starch. (**a**–**c**) were thermal properties of starch with varying contents of WB-E, WB-R, and WB layers, respectively. WB, wheat bran; AL, aleurone layer; NAL, non-aleurone layer; AL-E, extracts from AL-rich fraction; AL-R, residues of AL-rich fraction; NAL-E, extracts of NAL; NAL-R, residuals of NAL-rich fraction.

As shown in Figure 7 and Table S2, the addition of WB-E elevated the onset (T_o_), peak (T_p_), and conclusion (T_c_) temperatures of starch gelatinization, but significantly reduced ΔH (*p* < 0.05). This effect likely results from the negative impact of minerals on the plasticization capacity of water [20,31]. No significant differences in gelatinization temperature were observed between AL-E and NAL-E at equivalent concentrations. Both the extracts and residues significantly reduced ΔH, with the reduction becoming more pronounced at higher concentrations. WB exhibited a greater impact on starch gelatinization than its water-extracted residue at the same addition levels, suggesting that water extraction eliminates the solubilized components that negatively influence gelatinization [20].

**Figure 8:**
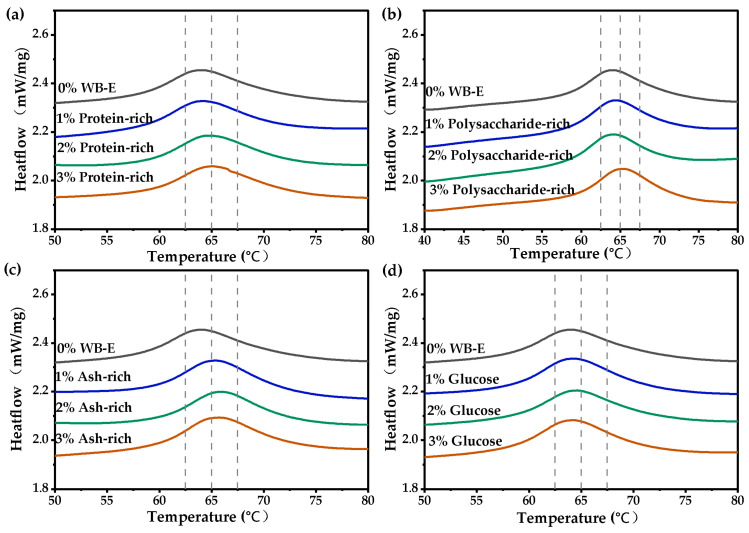
The influence of main components in WB-E on the thermal properties of wheat starch. WB-E, wheat bran extract. (**a**–**d**) were thermal properties of starch with varying contents of protein-rich, polysaccharide-rich, ash-rich and glucose fractions, respectively.

The influence of the different components in extracts on starch gelatinization were further investigated. As shown in Figure 8 and Table S3, at concentrations below 3%, the protein-rich fraction significantly reduced the ∆H, while the other components had no obvious effects. No significant differences were observed across the 1–3% dosage range. The addition of ash notably affected the T_o_, T_p_, and ∆H, while other components showed no significant influence on it. In theory, the minerals in the ash, such as potassium and magnesium, may prevent water molecules from entering the inside of starch granules, resulting in an incomplete gelatinization of starch [48,49]. Additionally, mineral ions entering the starch granules could modify the crystalline structure of starch, thereby reducing the gelatinization energy of starch (∆H) [47]. Soluble polysaccharides, such as AX, will compete with starch for water, inhibiting the gelatinization [10,11]. Similarly, proteins, mainly globulin and albumin in the extract, can restrict starch swelling and delay gelatinization [50]. However, at the tested concentrations, these components showed no significant effects, likely due to their limited quantities.

**Figure 9:**
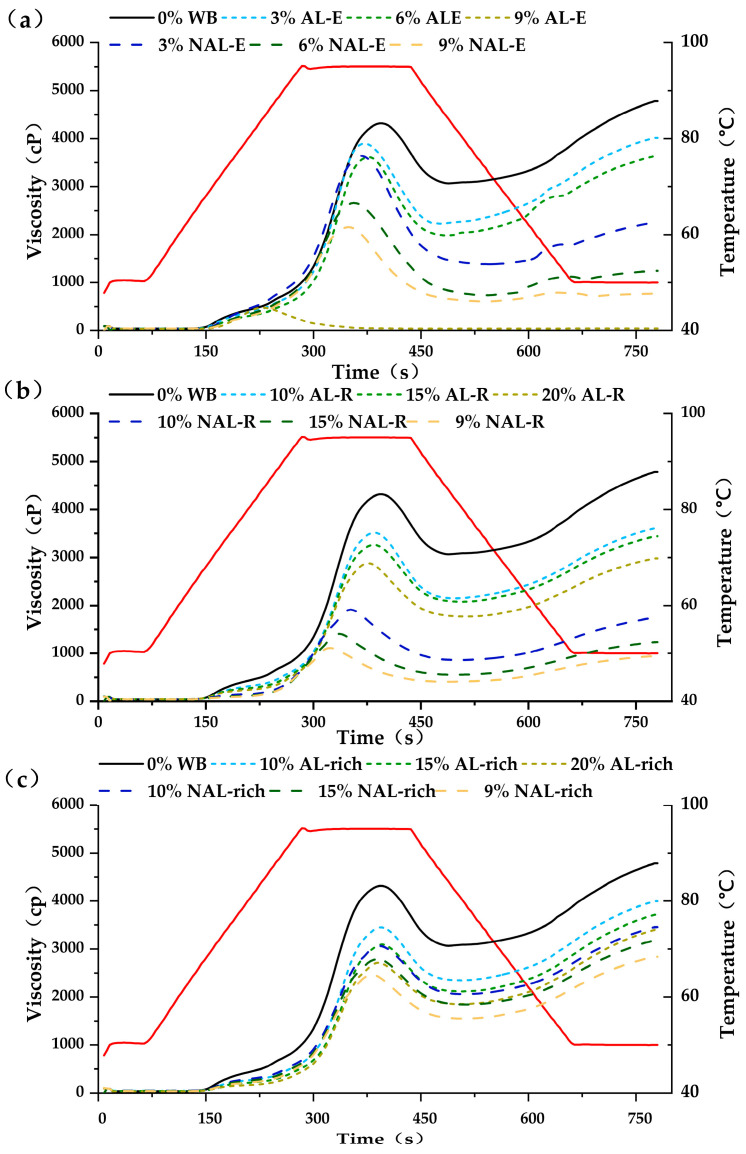
Influence of extracts and residuals from WB layers on the pasting property of wheat starch. (**a**–**c**) were pasting viscosity curves with varying contents of WB-E, WB-R, and WB layers, respectively.AL-rich, aleurone rich fraction; NAL-rich, non-aleurone layer rich fraction. AL-E extracts from AL-rich fraction; AL-R, residuals of AL-rich fraction after water extract; NAL-E, extracts of NAL; NAL-R, residuals of NAL-rich fraction after water extract. The red line in each graph represents the temperature profile, reflecting the programmed heating and cooling phases during the measurement.

Figure 9 and Table S4 show the effects of WB (AL-rich and NAL-rich), WB-E (AL-E and NAL-E), and WB-R (AL-R and NAL-R) on the pasting property of wheat starch. The addition of WB-E significantly altered starch gelatinization parameters (*p* < 0.05), reducing peak viscosity, final viscosity, and setback [51]. The results suggest that WB-E inhibits interactions between swelling starch granules, enhances thermal stability, and limits gel formation. Notably, starch pastes with the same amounts of AL-E and NAL-E showed notable different viscosities, with AL-E demonstrating higher values than NAL-E. This suggests that AL-E provided greater thermal resistance to starch granules than NAL-E [52]. Both WB-R (AL-R and NAL-R) and WB (AL-rich and NAL-rich) significantly reduced peak and final viscosities, as well as the setback. A previous study indicated that WB, especially the cell walls, could limit the viscosity of starch gel by competing with starch granules for water [53]. The high water retention of cell walls redistributed water molecules after pasting, inhibiting starch regeneration and slowing aging processes, ultimately reducing retrogradation [54]. Additionally, increasing WB or WB-R concentrations decreased the relative starch content in the system, leading to a lower final viscosity. The starch gel with NAL-E showed lower setback values than the AL-E, probably due to its higher content of non-polysaccharides (39%). Similarly, the starch gels with NAL-R and NAL showed lower setback values than their AL-R and AL counterparts.

**Figure 10:**
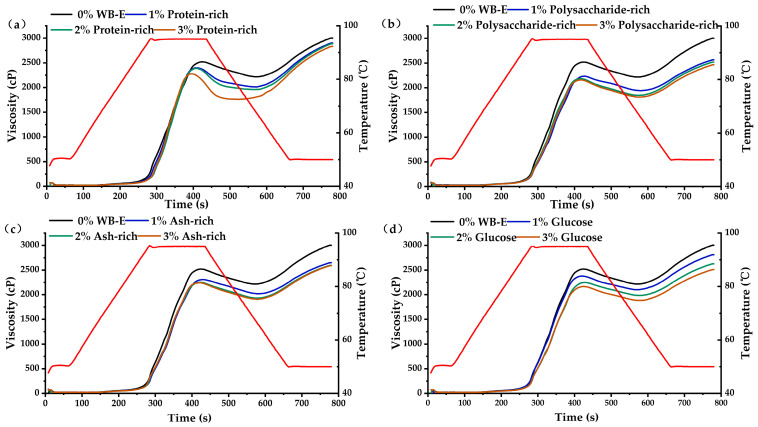
The influence of major components in WB-E on the pasting characteristics of starch. (**a**–**d**) were pasting viscosity curves with varying contents of protein-rich, polysaccharide-rich, ash-rich and glucose fractions, respectively. The red line in each graph represents the temperature profile, reflecting the programmed heating and cooling phases during the measurement.

The protein-rich, polysaccharides-rich, and ash-rich fractions from WB-E were added into wheat starch to elucidate their individual contributions to pasting properties. As shown in Figure 10 and Table S5, all the three components significantly reduced the peak viscosity and final viscosity of starch, consistent with findings by Teobaldi et al. [31]. In WB-E, the water-soluble AX and proteins (primarily globulin and albumin) can form hydrogen bonds or other non-covalent bonds with hydroxyl groups on the starch granule surface, limiting the starch swelling and movement of starch molecules, thus reducing the pasting viscosity of starch.

The polysaccharides from WB-E compete with starch for absorbing water, reducing the amount of free water available for starch gelatinization [55,56]. Additionally, they interact with starch granules to form a viscous matrix. Higher viscosity can hinder the leaching out of amylose from starch granules, thereby leading to cohesive starch gel and reduced retrogradation. The monosaccharides, primarily glucose and sucrose, lower the water activity and replace water molecules to form hydrogen bonds with starch molecules. Therefore, they will inhibit the gelatinization of starch.

Moreover, the proteins in the extract can also adsorb onto the surface of starch granules, creating a protein–starch complex that may decrease the leaching of amylose, making it more difficult to form a stable network structure during gelatinization and affecting the final texture of starch gels and their retrogradation [52,57]. In addition, both the ash and protein fractions decreased the setback dramatically. Salts can affect starch gelatinization based on their effect on water structure and electrostatic interaction with hydroxyl groups of starch. The effects depend on the type and concentration of the salt. According to the theory proposed by Jane J. L. [53], the salts that are structure-making will delay the migration of water molecules into the starch granule and increase the gelatinization temperature. In the extract from WB, K^+^, Mg^2+^, Ca^2+^, and P (mainly in the form of phytic acid) are the main minerals. These cations will delay the migration of water into the starch granule, limit starch gelatinization, and affect the retrogradation of starch and reduce the cold viscosity (low setback) of starch paste [50,54].

In summary, the proteins, polysaccharides, and ash components in WB-E collectively reduced starch pasting viscosity by restricting granule swelling via hydrogen bonding, inhibiting amylose leaching through non-covalent interactions, and suppressing retrogradation via ionic modulation of starch-water interactions.

## 4. Conclusions

This study investigates the differential impacts of water extracts from AL and NAL of wheat bran (WB) on the thermal and rheological properties of wheat gluten and starch. AL-E exhibited a higher extraction yield (30.6%) compared to NAL-E (15.1%), reflecting compositional disparities in proteins, minerals, and polysaccharides. For wheat gluten, the additions of extracts and residues from AL and NAL significantly reduced the denature temperature of wheat gluten, indicating their stabilizing effect on the gluten network. AL-E significantly reduced the G′ and G″ of gluten at addition levels of 3–9%. Conversely, NAL-E, with higher non-starch polysaccharides, initially increased G′ and G″ at 3–6%, but caused a sharp decline at 9%, suggesting a threshold-dependent interference with gluten hydration. AL-R and NAL-R increased gluten rigidity by physically hindering network development and competing for water, with AL-R showing stronger effects. The main components in the extract played different roles for the effects. The protein and ash fractions in the extract elevated the G′ and G″ of gluten at suitable dosages, while the polysaccharides deduced the G′ and G″ of gluten. The polysaccharide component at a high content (>2–3%) showed the negative impact of WB-E on gluten rheology.

As for starch properties, the aqueous extract from WB elevated gelatinization temperatures, but reduced enthalpy (ΔH). No significant differences in gelatinization temperature were observed between AL-E and NAL-E at equivalent concentrations. Ash notably affected the T_o_, T_p_, and ∆H, while the protein significantly reduced the ∆H. The polysaccharides showed no significant influence on it. Moreover, the addition of WB-E significantly reduced the peak viscosity, final viscosity, and setback. Starch pastes with the same amounts of AL-E and NAL-E showed notable different viscosities, with AL-E demonstrating higher values than NAL-E. The proteins, polysaccharide, and ash components in WB-E collectively reduced starch pasting viscosity by restricting granule swelling via hydrogen bonding, inhibiting amylose leaching through non-covalent interactions, and suppressing retrogradation via the ionic modulation of starch–water interactions.

These findings underscore the dual role of WB-E: while solubilized components (e.g., AX, minerals, proteins) impair gluten elasticity and starch viscosity, they concurrently enhance thermal stability and retard retrogradation. By contrast, WB-R exacerbates textural coarseness via insoluble fiber. The strategic fractionation of AL-E components (e.g., isolating minerals or AX) could enable the tailored modulation of dough properties, offering a pathway to improve whole-wheat product quality without compromising nutrition. This work provides a mechanistic foundation for optimizing WB utilization in gluten–starch systems. However, future research to be conducted within realistic food systems is needed. Such studies will bridge the gap between model system findings and practical applications, enabling the development of more effective strategies for utilizing WB in commercial food production.

## Figures and Tables

**Table 1:** Chemical composition of WB, AL-rich, and NAL-rich fractions obtained using TES.

Samples	Wheat Bran	AL-Rich Fraction	NAL-Rich Fraction
Water (%)	10.13 ± 0.05 ^a^	9.19 ± 0.05 ^b^	8.01 ± 0.23 ^c^
Starch (%)	13.08 ± 0.16 ^a^	3.58 ± 0.01 ^b^	2.13 ± 0.04 ^c^
Lipids (%)	3.22 ± 0.06 ^b^	5.41 ± 0.03 ^a^	2.98 ± 0.14 ^b^
Ash (%)	6.22 ± 0.02 ^b^	9.85 ± 0.04 ^a^	5.15 ± 0.04 ^c^
Phosphorus (%)	1.29 ± 0.00 ^b^	2.18 ± 0.04 ^a^	0.93 ± 0.01 ^c^
Protein (%)	16.56 ± 0.02 ^b^	20.13 ± 0.00 ^a^	11.86 ± 0.01 ^c^
Ferulic acid (mg/g)	4.150 ± 0.630 ^a^	5.077 ± 0.628 ^a^	3.924 ± 0.332 ^a^
*p*-coumaric acid (mg/g)	0.498 ± 0.056 ^a^	0.608 ± 0.017 ^a^	0.534 ± 0.033 ^a^
Proportion of AL (%)	44.9%	75.9%	32.4%

AL, aleurone layer; NAL, non-aleurone layer. The proportions of AL were calculated based on the content of phosphorus. Different letters in the same row denote significant differences between groups (*p* < 0.05).

**Table 2:** Composition and yield of the water extracts and residuals from different WB layers.

WB Layer Fractions	AL-Rich Fraction		NAL-Rich Fraction	
	AL-E	AL-R	NAL-E	NAL-R
Yield (%)	30.61 ± 0.79 ^c^	68.44 ± 1.16 ^b^	15.08 ± 0.45 ^d^	83.73 ± 0.74 ^a^
Water (%)	8.94 ± 0.22 ^b^	10.94 ± 0.03 ^a^	9.40 ± 0.17 ^b^	11.07 ± 0.09 ^a^
Ash (%)	19.51 ± 0.00 ^a^	5.85 ± 0.08 ^c^	16.09 ± 0.02 ^b^	2.77 ± 0.01 ^d^
Protein (%)	29.41 ± 0.43 ^a^	16.15 ± 0.32 ^c^	22.61 ± 0.85 ^b^	9.96 ± 0.28 ^d^
Lipids (%)	4.38 ± 0.02 ^b^	5.85 ± 0.14 ^a^	2.14 ± 0.03 ^d^	3.54 ± 0.09 ^c^
Non-Starch polysaccharide (%)	29.89 ± 0.21 ^d^	58.52 ± 0.11 ^b^	39.04 ± 0.18 ^c^	70.65 ± 0.15 ^a^
Phosphorus (%)	3.83 ± 0.01 ^a^	1.08 ± 0.05 ^c^	2.81 ± 0.01 ^b^	0.62 ± 0.01 ^d^

AL-E, extracts from AL-rich fraction; AL-R, residuals of AL-rich fraction; NAL-E, extracts of NAL; NAL-R, residuals of NAL-rich fraction. Different letters in the same row denote significant differences between groups (*p* < 0.05).

**Table 3:** Chemical composition of the major components in WB-E.

Composition	WB-E	Protein-Rich Fraction	Polysaccharide-Rich Fraction	Ash-Rich Fraction
Water (%)	23.69 ± 0.40 ^b^	6.34 ± 0.12 ^d^	10.16 ± 0.28 ^c^	29.89 ± 0.83 ^a^
Protein (%)	23.32 ± 0.0.26 ^c^	78.34 ± 0.54 ^a^	9.89 ± 0.48 ^d^	17.12 ± 0.29 ^b^
Ash (%)	19.25 ± 0.00 ^b^	1.84 ± 0.17 ^d^	18.06 ± 0.05 ^c^	32.35 ± 0.40 ^a^
Total carbohydrates (%)	31.70 ± 1.03 ^c^	13.10 ± 1.03 ^d^	53.44 ± 1.66 ^a^	20.51 ± 0.24 ^b^
Reducing sugar (%)	8.19 ± 0.22 ^b^	2.05 ± 0.07 ^c^	3.19 ± 0.09 ^c^	19.20 ± 0.64 ^a^

Different letters in the same row denote significant differences between groups (*p* < 0.05).

## Data Availability

The original contributions presented in this study are included in the article/Supplementary Materials. Further inquiries can be directed to the corresponding author.

## Supplementary Materials

The following supporting information can be downloaded at: https://www.mdpi.com/article/10.3390/foods14111988/s1, Table S1: Influence of extracts and residuals from WB layers on the thermal property of wheat gluten, Table S2: Influence of extracts and residues from WB layers on the thermal property of wheat starch, Table S3: The influence of main components in WB-E on thermal property of wheat starch, Table S4. Influence of extracts and residuals from WB layers on the pasting property of wheat starch, Table S5. The influence of major components in WB-E on the pasting characteristics of wheat starch.

## References

[1] Chen Z., Mense A.L., Brewer L.R., Shi Y. (2024). Wheat bran layers: Composition, structure, fractionation, and potential uses in foods. Crit. Rev. Food Sci. Nutr..

[2] Tu J., Liu G., Cao X., Zhu S., Li Q., Ji G., Han Y., Xiao H. (2019). Hypoglycemic effects of wheat bran alkyresorcinols in high-fat/high-sucrose diet and low-dose streptozotocin-induced type 2 diabetic male mice and protection of pancreatic β cells. Food Funct..

[3] Arslan M., Rakha A., Xiaobo Z., Mahmood M.A. (2019). Complimenting gluten free bakery products with dietary fiber: Opportunities and constraints. Trends Food Sci. Technol..

[4] Liu Y., Huang Y., Li L., Xiong Y., Tong L., Wang F., Fan B., Gong J. (2023). Effect of different agricultural conditions, practices, and processing on levels of total arsenic and species in cereals and vegetables: A review. Food Control.

[5] Pirhadi M., Shariatifar N., Bahmani M., Manouchehri A. (2022). Heavy metals in wheat grain and its impact on human health: A mini-review. J. Chem. Health Risks.

[6] Khalid K.H., Ohm J.B., Simsek S. (2017). Whole wheat bread: Effect of bran fractions on dough and end-product quality. J. Cereal Sci..

[7] Li C., Stump M., Wu W., Li Y. (2023). Exploring the chemical composition, antioxidant potential, and bread quality effects of the nutritional powerhouse: Wheat bran—A mini-review. J. Agric. Food Res..

[8] Antoine C., Peyron S., Mabille F., Lapierre C., Bouchet B., Abecassis J., Rouau X. (2003). Individual contribution of grain outer layers and their cell wall structure to the mechanical properties of wheat bran. J. Agric. Food Chem..

[9] Hemery Y., Rouau X., Lullien-Pellerin V., Barron C., Abecassis J. (2007). Dry processes to develop wheat fractions and products with enhanced nutritional quality. J. Cereal Sci..

[10] Stone B.A. (2006). Cell Walls of Cereal Grains. Cereal Foods World.

[11] Chen Z., Mense A.L., Brewer L.R., Shi Y.C. (2024). Wheat bran arabinoxylans: Chemical structure, extraction, properties, health benefits, and uses in foods. Compr. Rev. Food Sci. Food Saf..

[12] Liu W., Yang W., Wu J., Cheng Y., Wei Z., Wang T., Ampofo K.A., Ma H., Cui F., Yang X. (2021). ARTP mutagenesis to improve mycelial polysaccharide production of Grifola frondosa using a mixture of wheat bran and Rice bran as substrate. J. Food Qual..

[13] Prueckler M., Siebenhandl-Ehn S., Apprich S., Hoeltinger S., Haas C., Schmid E., Kneifel W. (2014). Wheat bran-based biorefinery 1: Composition of wheat bran and strategies of functionalization. LWT-Food Sci. Technol..

[14] He H., Qiao J., Liu Y., Guo Q., Ou X., Wang X. (2021). Isolation, structural, functional, and bioactive properties of cereal arabinoxylan—A critical review. J. Agric. Food Chem..

[15] Frederix S.A., Van Hoeymissen K.E., Courtin C.M., Delcour J.A. (2004). Water-extractable and water-unextractable arabinoxylans affect gluten agglomeration behavior during wheat flour gluten−starch separation. J. Agric. Food Chem..

[16] Wang P., Hou C., Zhao X., Tian M., Gu Z., Yang R. (2019). Molecular characterization of water-extractable arabinoxylan from wheat bran and its effect on the heat-induced polymerization of gluten and steamed bread quality. Food Hydrocoll..

[17] Balandrán-Quintana R.R., Mercado-Ruiz J.N., Mendoza-Wilson A.M. (2015). Wheat bran proteins: A review of their uses and potential. Food Rev. Int..

[18] Alzuwaid N.T., Pleming D., Fellows C.M., Sissons M. (2021). Fortification of durum wheat spaghetti and common wheat bread with wheat bran protein concentrate-impacts on nutrition and technological properties. Food Chem..

[19] Alzuwaid N.T., Sissons M., Laddomada B., Fellows C.M. (2019). Nutritional and functional properties of durum wheat bran protein concentrate. Cereal Chem..

[20] De Bondt Y., Liberloo I., Roye C., Goos P., Courtin C.M. (2020). The impact of wheat (*Triticum aestivum* L.) bran on wheat starch gelatinization: A differential scanning calorimetry study. Carbohydr. Polym..

[21] Yan J., Wu L., Cai W., Xiao G., Duan Y., Zhang H. (2019). Subcritical water extraction-based methods affect the physicochemical and functional properties of soluble dietary fibers from wheat bran. Food Chem..

[22] Qi Y., Yang Y., Hassane Hamadou A., Shen Q., Xu B. (2021). Tempering–preservation treatment inactivated lipase in wheat bran and retained phenolic compounds. Int. J. Food Sci. Technol..

[23] Mense A.L., Zhang C., Liu Q., Shi Y. (2024). Hydrothermal treatment and enzymatic hydrolysis of wheat bran and their effects on physical structure. ACS Food Sci. Technol..

[24] Li C., Chen G., Tilley M., Chen R., Perez-Fajardo M., Wu X., Li Y. (2024). Enhancing gluten network formation and bread-making performance of wheat flour using wheat bran aqueous extract. Foods.

[25] Wootton M., Shams-Ud-Din M. (1986). The effects of aqueous extraction on the performance of wheat bran in bread. J. Sci. Food Agric..

[26] Obadi M., Zhang J., Shi Y., Xu B. (2021). Factors affecting frozen cooked noodle quality: A review. Trends Food Sci. Technol..

[27] Li Q., Li C., Li E., Gilbert R.G., Xu B. (2020). A molecular explanation of wheat starch physicochemical properties related to noodle eating quality. Food Hydrocoll..

[28] An D., Li H., Li D., Zhang D., Huang Y., Obadi M., Xu B. (2022). The relation between wheat starch properties and noodle springiness: From the view of microstructure quantitative analysis of gluten-based network. Food Chem..

[29] Chen Z., Xia Q., Zha B., Sun J., Xu B., Chen Z. (2020). Triboelectric separation of wheat bran tissues: Influence of tribo–material, water content, and particle size. J. Food Process Eng..

[30] Chen Z., Wang L., Wang R., Li Y., Chen Z. (2014). Triboelectric separation of aleurone cell-cluster from wheat bran fragments in nonuniform electric field. Food Res. Int..

[31] Teobaldi A.G., Barrera G.N., Ribotta P.D. (2024). Effect of damaged starch and wheat-bran arabinoxylans on wheat starch and wheat starch–gluten systems. Foods.

[32] American Association of Cereal Chemists (2009). Approved Methods of the AACC.

[33] Association of Official Analytical Chemists (2012). Official Methods of Analysis of AOAC International.

[34] Nielsen S.S., Nielsen S.S. (2009). Phenol-sulfuric acid method for total carbohydrates. Food Analysis Laboratory Manual.

[35] Jain A., Jain R., Jain S., Jain A., Jain R., Jain S. (2020). Quantitative analysis of reducing sugars by 3, 5-dinitrosalicylic acid (DNSA method). Basic Techniques in Biochemistry, Microbiology and Molecular Biology.

[36] Antoine C., Peyron S., Lullien-Pellerin V., Abecassis J., Rouau X. (2004). Wheat bran tissue fractionation using biochemical markers. J. Cereal Sci..

[37] Dobberstein D., Bunzel M. (2010). Separation and detection of cell wall-bound ferulic acid dehydrodimers and dehydrotrimers in cereals and other plant materials by reversed phase high-performance liquid chromatography with ultraviolet detection. J. Agric. Food Chem..

[38] Chen Z., Shen J., Yang Y., Wang H., Xu B. (2022). Intact aleurone cells limit the hydrolysis of endogenous lipids in wheat bran during storage. Food Res. Int..

[39] Luna-Valdez J., Balandrán-Quintana R., Azamar-Barrios J., Clamont-Montfort G.R., Mendoza-Wilson A., Mercado-Ruiz J., Madera-Santana T., Rascon-Chu A., Chaquilla-Quilca G. (2017). Structural and physicochemical characterization of nanoparticles synthesized from an aqueous extract of wheat bran by a cold-set gelation/desolvation approach. Food Hydrocoll..

[40] Wang M., Hamer R.J., Vliet T.V., Gruppen H., Marseille H., Weegels P.L. (2002). Effect of water unextractable solids on gluten formation and properties: Mechanistic considerations. J. Cereal Sci..

[41] Noort M.W.J., Haaster D.V., Hemery Y., Schols H.A., Hamer R.J. (2010). The effect of particle size of wheat bran fractions on bread quality—Evidence for fibre–protein interactions. J. Cereal Sci..

[42] Li H.-T., Kerr E.D., Schulz B.L., Gidley M.J., Dhital S. (2022). Pasting properties of high-amylose wheat in conventional and high-temperature Rapid Visco Analyzer: Molecular contribution of starch and gluten proteins. Food Hydrocoll..

[43] Turner M.A., Soh C.H., Ganguli N.K., Sissons M. (2008). A survey of water–extractable arabinopolymers in bread and durum wheat and the effect of water-extractable arabinoxylan on durum dough rheology and spaghetti cooking quality. J. Sci. Food Agric..

[44] Labat E., Rouau X., Morel M.-H. (2002). Effect of flour water-extractable pentosans on molecular associations in gluten during mixing. LWT-Food Sci. Technol..

[45] Liu S., Li Y., Obadi M., Jiang Y., Chen Z., Jiang S., Xu B. (2019). Effect of steaming and defatting treatments of oats on the processing and eating quality of noodles with a high oat flour content. J. Cereal Sci..

[46] Wang F., Chao H., Xu Z., Wu Y., Sun L., Wang N. (2022). Bran characteristics impact the whole wheat noodle quality. Food Sci. Technol..

[47] Miller R., Hoseney R. (2008). Role of salt in baking. Cereal Foods World.

[48] Tester R., Sommerville M. (2003). The effects of non-starch polysaccharides on the extent of gelatinisation, swelling and α-amylase hydrolysis of maize and wheat starches. Food Hydrocoll..

[49] Yang C., Zhong F., Douglas Goff H., Li Y. (2019). Study on starch-protein interactions and their effects on physicochemical and digestible properties of the blends. Food Chem..

[50] Baxter G., Blanchard C., Zhao J. (2014). Effects of glutelin and globulin on the physicochemical properties of rice starch and flour. J. Cereal Sci..

[51] Li Q., Liu S., Obadi M., Jiang Y., Zhao F., Jiang S., Xu B. (2020). The impact of starch degradation induced by pre-gelatinization treatment on the quality of noodles. Food Chem..

[52] Miaomiao S., Xuena D., Yanqiu C., Xiaolong J., Yanqi L., Yizhe Y. (2023). Preparation and characterization of extruded yam starch–soy protein isolate complexes and their effects on the quality of dough. Foods.

[53] Jane J.L. (2006). Mechanism of Starch Gelatinization in Neutral Salt Solutions. Starch-Stärke.

[54] Samutsri W., Suphantharika M. (2012). Effect of salts on pasting, thermal, and rheological properties of rice starch in the presence of non-ionic and ionic hydrocolloids. Carbohydr. Polym..

[55] Zhou T., Zhang Y., Zhou S., Liu Q., Lin Q., Wang Y., Yang Y., Qiu C., Jiao A., Jin Z. (2025). The influence of water-soluble arabinoxylan on the rheological properties of model dough and the interfacial properties of imitation dough liquor. Food Chem..

[56] Kang J., Huang-Fu Z.-Y., Tian X., Cheng L., Zhang J., Liu Y., Liu Y., Wang S., Hu X., Zou L. (2023). Arabinoxylan of varied structural features distinctively affects the functional and in vitro digestibility of wheat starch. Food Hydrocoll..

[57] Pi X., Zhu L., Xiang M., Zhao S., Cheng Z., Qiao D., Zhang B. (2025). Insight of soy protein isolate to decrease the gel properties corn starch based binary system: Rheological and structural investigation. Food Hydrocoll..

