# Supplementary material for: Effects of Aqueous Extracts from Wheat Bran Layers on the Functional Properties of Wheat Starch and Gluten
Source: Foods. 2025 Jun 4;14(11):1988. doi: 10.3390/foods14111988 (PMC12155330; doi:10.3390/foods14111988)
Supplement: Supplementary file 1 [file foods-14-01988-s001.zip › foods-3605715-SI.pdf]

Supplement materials

Table S1. Influence of extracts and residuals from WB layers on the thermal property of wheat gluten.

Addition amount (%)	Denaturation temperature (°C)	ΔH (J/g)
0% WB	58.57±0.33[a]	1.43 ± 0.01[b]
3% AL-E	49.08 ± 0.57[cd]	1.54 ± 0.17[ab]
6% AL-E	48.93 ± 0.13[cd]	1.44 ± 0.22[b]
9% AL-E	49.59 ± 0.91[cd]	1.54 ± 0.29[a]
3% NAL-E	50.32 ± 0.25[c]	1.59 ± 0.09[ab]
6% NAL-E	53.56 ± 0.86[b]	1.88 ± 0.06[a]
9% NAL-E	48.91 ± 0.25[d]	1.68 ± 0.06[ab]
10% AL-R	57.06 ± 0.25[ab]	1.81 ± 0.33[ab]
15% AL-R	56.26 ± 0.45[b]	1.41 ± 0.13[bc]
20% AL-R	54.10 ± 2.20[c]	1.18 ± 0.07[c]
10% NAL-R	56.78 ± 0.28[ab]	2.12 ± 0.27[a]
15%NAL-R	53.67 ± 0.33[c]	1.20 ± 0.07[c]
20%NAL-R	49.82 ± 0.07[d]	1.22 ± 0.17[c]
10% AL-rich	50.26 ± 0.35[b]	1.47 ± 0.03[b]
15% AL-rich	52.12 ± 0.35[b]	1.61 ± 0.26[ab]
20% AL-rich	49.59 ± 0.02[d]	1.20 ± 0.04[b]
10% NAL-rich	49.90 ± 0.18[cd]	2.09 ± 0.54[a]
15% NAL-rich	50.33 ± 0.11[b]	1.54 ± 0.04[ab]
20% NAL-rich	50.26 ± 0.13[b]	1.42 ± 0.13[b]

AL, Aleurone layer; NAL, Non Aleurone layer; AL-E, extracts from AL-rich fraction; AL-R, residues of AL-rich fraction; NAL-E, extracts of NAL; NAL-R, residuals of NAL-rich fraction. Each group within the same column (WB-E, WB-R, and WB layers) was statistically compared to the 0% WB. Different superscript lowercase letters indicate significant differences ($p < 0.05$) among parameters. 0–9% AL-E, dough made with gluten and 0–9% AL-E addition; 0–9% NAL-E, dough made with gluten and 0–9% NAL-E addition; 0–20% AL-R, dough made with gluten and 0–20% AL-R addition; 0–20% NAL-R, dough made with gluten and 0–20% NAL-R addition; 0–20% AL-rich, dough made with gluten and 0–20% AL-rich fraction addition; 0–20% NAL, dough made with gluten and 0–20% NAL-rich fraction addition.

Table S2. Influence of extracts and residues from WB layers on the thermal property of wheat starch.

Addition amount (%)	Onset (°C)	Peak (°C)	End (°C)	ΔH (J/g)
0% WB	58.55 ± 0.17^{d}	63.89 ± 0.05^{d}	70.93 ± 1.34^{b}	8.95 ± 0.01^{a}
3% AL-E	60.51 ± 0.2^{bc}	65.75 ± 0.28^{c}	72.84 ± 0.38^{a}	8.62 ± 0.00^{a}
6% AL-E	60.66 ± 0.11^{bc}	65.98 ± 0.07^{c}	72.99 ± 0.54^{a}	8.49 ± 0.56^{a}
9% AL-E	60.79 ± 0.5^{bc}	66.20 ± 0.29^{bc}	72.89 ± 0.15^{a}	5.79 ± 0.36^{d}
3% NAL-E	60.57 ± 0.02^{bc}	65.87 ± 0.15^{c}	72.86 ± 0.32^{a}	7.82 ± 0.06^{b}
6% NAL-E	60.24 ± 1.79^{cd}	65.34 ± 1.53^{c}	72.22 ± 0.54^{ab}	6.65 ± 0.14^{c}
9% NAL-E	62.39 ± 0.72^{b}	67.52 ± 0.16^{b}	72.89 ± 0.15^{a}	5.99 ± 0.21^{d}
10% AL-R	60.75 ± 0.25^{c}	65.66 ± 0.35^{bc}	72.18 ± 0.77^{b}	5.27 ± 0.05^{bc}
15% AL-R	61.05 ± 0.29^{bc}	65.78 ± 0.18^{bc}	71.62 ± 0.07^{b}	4.36 ± 0.33^{de}
20% AL-R	61.67 ± 0.16^{b}	66.19 ± 0.35^{b}	72.39 ± 0.52^{b}	4.13 ± 0.04^{e}
10% NAL-R	60.74 ± 0.57^{c}	65.41 ± 0.37^{c}	71.81 ± 0.91^{b}	5.53 ± 0.51^{b}
15%NAL-R	61.34 ± 0.12^{bc}	65.81 ± 0.24^{bc}	71.99 ± 0.05^{b}	4.87 ± 0.31^{cd}
20%NAL-R	61.35 ± 0.21^{bc}	65.77 ± 0.16^{bc}	71.88 ± 0.45^{b}	3.89 ± 0.47^{e}
10% AL-rich	61.34 ± 0.27^{c}	66.1 ± 0.13^{bc}	72.91 ± 0.09^{a}	6.23 ± 0.37^{b}
15% AL-rich	62.12 ± 0.01^{b}	66.73 ± 0.06^{ab}	73.14 ± 0.55^{a}	5.70 ± 1.23^{bc}
20% AL-rich	62.87 ± 0.03^{a}	67.14 ± 0.31^{a}	73.18 ± 0.39^{a}	5.00 ± 0.34^{bc}
10% NAL-rich	61.23 ± 0.29^{c}	65.74 ± 0.01^{c}	72.46 ± 0.01^{ab}	5.87 ± 0.18^{b}
15% NAL-rich	62.13 ± 0.18^{b}	66.14 ± 0.13^{bc}	72.52 ± 0.43^{ab}	5.33 ± 0.05^{bc}
20% NAL-rich	62.18 ± 0.35^{b}	66.13 ± 0.63^{bc}	72.24 ± 0.86^{ab}	4.41 ± 0.52^{c}

Each group within the same column (AL-E, NAL-E, AL-R, NAL-R, AL-rich and NAL-rich) was statistically compared to the 0% WB. Different superscript lowercase letters indicate significant differences ($p < 0.05$) among parameters. WB-E: AL-E and NAL-E; WB-R: AL-R and NAL-R. WB layers-rich: AL-rich and NAL-rich.

Table S3. The influence of main components in WB-E on thermal property of wheat starch.

Addition amount (%)	Onset (℃)	Peak (℃)	End (℃)	ΔH (J/g)
0% WB-E	58.59 ± 0.24^{b}	64.18 ± 0.11^{b}	72.34 ± 0.06^{ab}	7.88 ± 0.6^{a}
1% Protein-rich	58.91 ± 0.01^{bc}	64.17 ± 0.22^{c}	71.74 ± 0.11^{a}	7.00 ± 0.19^{ab}
2% Protein-rich	58.57 ± 0.5^{c}	64.51 ± 0.45^{bc}	71.82 ± 1.32^{a}	6.74 ± 0.11^{b}
3% Protein-rich	59.60± 0.06^{b}	65.11 ± 0.05^{b}	71.06 ± 2.52^{a}	6.60± 0.26^{b}
1% polysaccharide-rich	58.74 ± 0.11^{b}	64.37 ± 0.06^{b}	71.20 ± 0.52^{b}	7.74 ± 0.36^{a}
2% polysaccharide-rich	58.68 ± 0.45^{b}	64.19 ± 0.28^{b}	71.32 ± 0.49^{b}	7.29 ± 0.41^{a}
3% polysaccharide-rich	59.93 ± 0.13^{b}	65.54 ± 0.29^{b}	73.02 ± 0.58^{a}	7.46 ± 0.07^{a}
1% Ash-rich	59.22 ± 0.65^{ab}	64.91 ± 0.64^{ab}	72.47 ± 0.76^{a}	7.45 ± 0.06^{a}
2% Ash-rich	60.05 ± 0.39^{a}	65.75 ± 0.38^{a}	73.32 ± 0.71^{a}	7.08 ± 0.33^{a}
3% Ash-rich	59.93 ± 0.46^{a}	65.27 ± 0.66^{ab}	72.47 ± 0.65^{a}	7.21 ± 0.29^{a}
1% Glucose	58.74 ± 0.05^{b}	64.14 ± 0.36^{b}	71.96 ± 0.34^{a}	7.98 ± 0.23^{a}
2% Glucose	58.81 ± 0.23^{b}	64.26 ± 0.45^{b}	72.05 ± 0.23^{a}	7.62 ± 0.08^{a}
3% Glucose	59.10 ± 0.35^{b}	64.44 ± 0.41^{b}	71.93 ± 0.68^{a}	7.35 ± 0.02^{a}

Each group within the same column (Protein-rich, polysaccharide-rich, Ash-rich, Glucose) was statistically compared to the 0% WB-E. Different superscript lowercase letters indicate significant differences ($p < 0.05$) among parameters.

Table S4. Influence of extracts and residuals from WB layers on the pasting property of wheat starch.

Addition amount (%)	Pasting time (min)	Peak viscosity (cP)	Trough (cP)	Breakdown (cP)	Final viscosity (cP)	Setback (cP)
0% WB-E	7.00±0.00^{a}	5537.5 ± 111.5^{a}	4438.5 ± 151.5^{a}	1099.0 ± 40.0^{c}	7026.5 ± 108.5^{a}	2588.0 ± 43.0^{a}
3% AL-E	7.00±0.00^{a}	5136.0 ± 2.0^{b}	4005.0 ± 34.0^{b}	1131.0 ± 32.0^{c}	6333.5 ± 1.5^{b}	2328.5 ± 35.5^{b}
6% AL-E	7.00±0.00^{a}	4899.5 ± 29.5^{b}	3594.5 ± 109.5^{c}	1305.0 ± 80.0^{b}	5689.0 ± 37.0^{d}	2094.5 ± 72.5^{c}
9% AL-E	6.93±0.00^{b}	3860.5 ± 6.4^{c}	2734.5 ± 40.3^{d}	1126 ± 46.7^{c}	4429.5 ± 9.2^{f}	1695 ± 31.1^{d}
3% NAL-E	7.00±0.00^{a}	5084.5 ± 16.5^{b}	4130.5 ± 10.5^{b}	954.0 ± 6.0^{d}	6150.0 ± 15.0^{c}	2019.5 ± 25.5^{c}
6% NAL-E	6.93±0.00^{b}	4271.0 ± 69.3^{c}	3425.5 ± 74.2^{c}	845.5 ± 4.9^{d}	4815 ± 111.7^{e}	1389.5 ± 37.5^{e}
9% NAL-E	6.10±0.05^{c}	2766.5 ± 57.3^{d}	1162 ± 17.0^{e}	1604.5 ± 40.3^{a}	1791.5 ± 47.4^{g}	629.5 ± 30.4^{f}
10% AL-R	7.00±0.00^{a}	4264.5 ± 7.8^{b}	3402.0 ± 2.8^{b}	862.5 ± 4.9^{d}	5056.5 ± 12.0^{b}	1654.5 ± 9.2^{b}
15% AL-R	7.00±0.00^{a}	4025.5 ± 7.8^{b}	3213.5 ± 65.8^{bc}	812.0 ± 73.5^{d}	4766.5 ± 43.1^{c}	1553.0 ± 22.6^{c}
20% AL-R	6.93±0.00^{b}	3701.0 ± 35.4^{c}	3028.5 ± 9.2^{d}	672.5 ± 26.2^{e}	4304.5 ± 36.1^{d}	1276.0 ± 26.9^{d}
10% NAL-R	5.93±0.00^{c}	3196.0 ± 100.4^{d}	1610.5 ± 82.7^{e}	1585.5 ± 17.7^{a}	2568.0 ± 100.4^{e}	957.5 ± 17.7^{e}
15% NAL-R	5.60±0.00^{d}	2491.5 ± 9.2^{e}	968.5 ± 2.1^{e}	1523.0 ± 7.1^{a}	1719.0 ± 4.2^{f}	750.5 ± 6.4^{f}
20% NAL-R	5.50±0.05^{e}	1958.5 ± 20.5^{f}	686.0 ± 11.3^{f}	1272.5 ± 9.2^{b}	1271.5 ± 14.8^{g}	585.5 ± 3.5^{g}
10% AL-rich	6.97±0.05^{a}	4820.5 ± 106.8^{b}	3258.0 ± 181.0^{bc}	1562.5 ± 287.8^{bc}	5736.5 ± 54.4^{b}	2478.5 ± 235.5^{ab}
15% AL-rich	6.87±0.19^{ab}	4593.5 ± 136.5^{c}	3028.5 ± 608.8^{bc}	1565.0 ± 472.3^{bc}	5415.0 ± 158.4^{c}	2386.5 ± 450.4^{ab}
20% AL-rich	6.78±0.07^{ab}	3838.0 ± 4.2^{e}	2637.0 ± 332.3^{c}	1201.0 ± 328.1^{c}	4766.0 ± 50.9^{d}	2129.0 ± 281.4^{abc}
10% NAL-rich	6.87±0.09^{ab}	4517 ± 18.4^{c}	3481.5 ± 130.8^{b}	1035.5 ± 112.4^{c}	5429.0 ± 56.6^{c}	1947.5 ± 74.2^{bc}
15% NAL-rich	6.83±0.05^{ab}	4163 ± 43.8^{d}	3167.5 ± 92.6^{bc}	995.5 ± 136.5^{c}	4937.0 ± 18.4^{d}	1769.5 ± 74.2^{c}
20% NAL-rich	6.67±0.00^{b}	3657.5 ± 40.3^{e}	2846.0 ± 35.4^{bc}	811.5 ± 4.9^{cd}	4452.0 ± 15.6^{e}	1606.0 ± 19.8c

AL-rich, aleurone rich fraction; NAL-rich, non-aleurone layer rich fraction. AL-E, extracts from AL-rich fraction; AL-R, residuals of AL-rich fraction after water extract; NAL-E, extracts of NAL; NAL-R, residuals of NAL-rich fraction after water extract. Each group (WB-E, AL-E, NAL-E, AL-R, NAL-R, AL-rich and NAL-rich) within the same column was statistically compared to the 0% WB. Different superscript lowercase letters indicate significant differences ($p < 0.05$) in the same column.

Table S5. The influence of major components in WB-E on the pasting characteristics of wheat starch.

Addition amount (%)	Pasting time (min)	Peak viscosity (cP)	Trough (cP)	Breakdown (cP)	Final viscosity (cP)	Setback (cP)
0% WB-E	7.00±0.00a	2528.5 ± 14.8a	1881.0 ± 22.6a	647.5 ± 37.5a	2980.0 ± 32.5a	1099.0 ± 9.9a
1% Protein-rich	6.83±0.05b	2257.5 ± 44.5b	1600.0 ± 33.9c	657.5 ± 10.6a	2600.0 ± 48.1b	1000.0 ± 14.1ab
2% Protein-rich	6.73±0.09bc	2246.0 ± 89.1b	1696.5 ± 9.2b	549.5 ± 79.9ab	2600.0 ± 111.7b	903.5 ± 102.5bc
3% Protein-rich	6.63±0.05c	2098.0 ± 82.0b	1593.0 ± 45.3c	505.0 ± 36.8c	2392.5 ± 102.5b	799.5 ± 57.3c
1% Polysaccharide-rich	7.00±0.00 a	2425.0 ± 35.4b	1908.5 ± 3.5ab	516.5 ± 38.9b	2944.5 ± 62.9a	1036.0 ±66.5a
2% Polysaccharide-rich	6.98±0.01b	2388.0 ± 1.4b	1899.5 ± 53ab	488.5 ± 54.4b	2915.5 ± 41.7ab	1016.0 ± 11.3a
3% Polysaccharide-rich	6.93±0.00c	2287.0 ± 9.9c	1797.0 ± 49.5b	490.0 ± 39.6b	2820.5 ± 14.8b	1023.5 ± 64.3a
1% Ash-rich	7.00±0.00 a	2359.5 ± 85.6ab	1726.5 ± 67.2ab	633.0 ± 18.4a	2726.0 ± 110.3ab	999.5 ± 43.1ab
2% Ash-rich	7.00±0.00 a	2300.0 ± 66.5ab	1732.0 ± 41.0ab	568.0 ± 25.5ab	2656.0 ± 87.7ab	924.0 ± 46.7bc
3% Ash-rich	7.00±0.00 a	2141.5 ± 143.5b	1634.0 ± 103.2b	507.5 ± 40.3b	2454.5 ± 190.2b	820.5 ± 87.0c
1% Glucose	7.00±0.00 a	2425.5 ± 64.3a	1825.0 ± 4.2a	600.5 ± 68.6a	2848.5 ± 54.4a	1023.5 ± 58.7ab
2% Glucose	7.00±0.00 a	2276.0 ± 46.7b	1647.0 ± 60.8b	629.0 ± 14.1a	2666.5 ± 55.9b	1019.5 ± 4.9ab
3% Glucose	7.00±0.00 a	2176.0 ± 17.0b	1609.0 ± 7.1b	567.0 ± 24.0a	2544.0 ± 49.5b	935.0 ± 56.6b

Each group within the same column (Protein-rich, polysaccharide-rich, Ash-rich and WB layers) was statistically compared to the 0% WB-E. Different superscript lowercase letters indicate significant differences ($p < 0.05$) among parameters.
